# Separable networks for top-down attention to auditory non-spatial and visuospatial modalities

**DOI:** 10.1016/j.neuroimage.2013.02.023

**Published:** 2013-07-01

**Authors:** Rodrigo M. Braga, Liam R. Wilson, David J. Sharp, Richard J.S. Wise, Robert Leech

**Affiliations:** a Computational, Cognitive and Clinical Neuroimaging Laboratory C3NL Burlington Danes Building, Hammersmith Hospital Campus, Du Cane Road, Imperial College London, London W12 0NN, UK; b MRC Clinical Sciences Centre, Faculty of Medicine, Imperial College London, Hammersmith Hospital Campus, London W12 0NN, UK

**Keywords:** DAN, Dorsal attention network, MFG, Middle frontal gyrus, IFG, Inferior frontal gyrus, ITG, Inferior temporal gyrus, SPL, Superior parietal lobe, FEF, Frontal eye fields, pMTG, Posterior middle temporal gyrus, VAN, Ventral attention network, fMRI, Functional magnetic resonance imaging, BOLD, Blood oxygenation level dependent, ICA, Independent component analysis, A_p_, Attentive phase, P_p_, Passive phase, Top-down attention, Visual, Auditory, Dorsal attention network, Independent component analysis, Endogenous

## Abstract

A central question for cognitive neuroscience is whether there is a single neural system controlling the allocation of attention. A dorsal frontoparietal network of brain regions is often proposed as a mediator of top-down attention to all sensory inputs. We used functional magnetic resonance imaging in humans to show that the cortical networks supporting top-down attention are in fact modality-specific, with distinct superior fronto-parietal and fronto-temporal networks for visuospatial and non-spatial auditory attention respectively. In contrast, parts of the right middle and inferior frontal gyri showed a common response to attentional control regardless of modality, providing evidence that the amodal component of attention is restricted to the anterior cortex.

## Highlights

► Fronto-temporal systems involved in auditory non-spatial top-down attention ► Dorsal attention network involved in visuospatial top-down attention ► Only middle and inferior frontal gyri significantly activated for both modalities ► There are modality specific neural mechanisms modulating top-down attention

## Introduction

The ability to select task-relevant information (top-down or endogenous attention) is central to high-level cognition, perception and behavior (Posner and Petersen, 1990). The assumption that there is a single system mediating top-down attention to all sensory modalities underlies many theoretical accounts of cognitive control (Corbetta et al., 2008, Posner and Petersen, 1990, Spence and Driver, 1997). A frontoparietal network that includes the superior parietal lobe (SPL), frontal eye fields (FEF) and middle frontal gyrus (MFG) is activated during many studies of top-down attention (Kincade et al., 2005, Vossel et al., 2006) and has been labeled the “dorsal attentional network” (DAN; Corbetta et al., 2008). In contrast, a more inferior network that includes the MFG and temporoparietal junction (the “ventral attention network” or VAN) is activated together with the DAN when attention is captured by behaviorally relevant stimuli (bottom-up or exogenous attention), in what has been termed the ‘reorienting response’ (Corbetta et al., 2008).

The DAN is widely assumed to be amodal, supporting top-down attention to visual, auditory and somatosensory inputs (Driver and Spence, 1998, Langner et al., 2011, Macaluso, 2010, Posner and Petersen, 1990). However, the evidence for this network comes overwhelmingly from visual studies (Corbetta et al., 2008), which agree with reports that the SPL and FEF are strongly involved in visuospatial processing (Behrmann, 2004) and controlling eye movement (Büttner-Ennever and Horn, 1997). For example, the FEF and SPL have been shown to have a strong retinotopic organization both with direct stimulation and functional neuroimaging (Moore et al., 2003, Ruff et al., 2008, Saygin and Sereno, 2008). In vision, both spatial and non-spatial attention tasks have implicated the SPL and FEF (Marois et al., 2000). However, in audition, Shomstein and Yantis (2006) found activation of the SPL but not FEF during spatial attention, and reported SPL deactivation during non-spatial sections of the task. Therefore, although DAN involvement in visual attention is supported by neuropsychological, retinotopic and oculomotor studies, it is less clear whether two core nodes of the DAN, the FEF and SPL, are needed for attending to other sensory modalities such as audition.

Previous functional imaging studies have implicated the full DAN in processing auditory stimuli (Davis et al., 2000, Driver and Spence, 1998, Hallett et al., 1999, Langner et al., 2011, Linden, 1999, Macaluso et al., 2003, Maeder et al., 2001, Mayer et al., 2006, Shomstein and Yantis, 2006, Sridharan et al., 2007, Wu et al., 2007). However, many of these studies focused on crossmodal attention, in which attention to each modality alone cannot be sufficiently separated. For instance, papers that presented visual stimuli to cue auditory attention (Davis et al., 2000, Driver and Spence, 1998, Langner et al., 2011, Macaluso et al., 2003) cannot exclude the effects of visual processing from auditory top-down attention. Along similar lines, papers that analyzed the period when auditory targets were actually displayed (Linden, 1999, Maeder et al., 2001, Mayer et al., 2006, Shomstein and Yantis, 2006, Sridharan et al., 2007) cannot be said to be looking only at top-down attention, as bottom up and executive networks would be elicited by the presentation of the target. Other papers included an immediate button response to a target (Langner et al., 2011, Mayer et al., 2006, Shomstein and Yantis, 2006) and therefore cannot dissociate the effects of the preparation for and execution of a motor response. These are significant confounds which might evoke DAN activation due to visual or spatial causes. These issues are particularly problematic in studies that use rapid trial times (< 5 s; Davis et al., 2000, Hallett et al., 1999, Langner et al., 2011, Macaluso et al., 2003, Maeder et al., 2001, Mayer et al., 2006, Wu et al., 2007, Zatorre et al., 1999), where activations for cues, targets and motor responses are difficult to separate due to the hemodynamic lag. It is therefore hard to say that the previous studies suitably isolated the networks for top-down auditory attention from spatial, crossmodal and executive confounds.

When functional imaging studies have focused on the auditory processing of speech and music, DAN activation is rarely observed. For example, a meta-analysis of 128 language studies showed no activation peaks within SPL and FEF during auditory processing of speech (Vigneau et al., 2011); and see also Cabeza and Nyberg (2000). Similarly, the DAN is not typically observed in studies of music processing (Hickok et al., 2003, Warren, 2008). The neuropsychological evidence also does not support an amodal DAN. Focal parietal lesions which lead to visuospatial neglect (Malhotra et al., 2009) often do not lead to deficits in detecting or identifying sounds, although auditory spatial localization (Pavani et al., 2002) and sustained attention deficits have been reported (Robertson et al., 1997). This suggests that parietal lobe neglect predominantly affects spatial and visual modalities. Hence, although there is compelling evidence for DAN involvement in top-down visuospatial attention, the evidence that the full SPL–FEF–MFG axis is necessary for auditory attention is inconclusive.

## Materials and methods

**Fig. 1:**
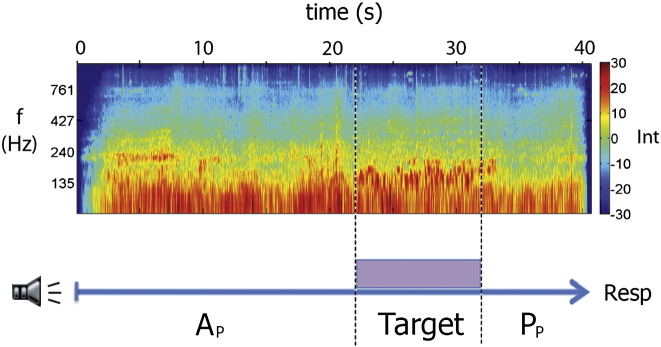
Auditory search task design. Background sounds (spectrogram and blue arrow) were divided into attentive (A_p_) and passive (P_p_) listening phases by the presence of a 10 s target foreground sound. The extended trial duration (40 s) allowed the attentional state during A_p_ and P_p_ to be clearly separated from target and button response (Resp) evoked activations. The auditory input was equivalent during Ap and P_p_. Int: Intensity, f: frequency.

We used functional magnetic resonance imaging (fMRI) to identify networks active during auditory top-down attention in the absence of visual or spatial requirements. A simple non-spatial auditory search task was used (see Fig. 1). Subjects listened to complex natural background sounds and were instructed to listen out for a pitch change that occurred within a pre-trained target sound. The presence of a target divided each trial into three phases: (1) an extended active listening phase (A_p_), where subjects listened to the background auditory scenes in order to detect the target sound; (2) a target phase, during which subjects were required to listen to the target and identify whether it contained a pitch change; and (3) a post-target passive listening phase (P_p_), where subjects heard the background sounds but had no requirement to listen attentively. Once subjects identified a target they were aware that there was no requirement to listen attentively. We compared the neural activity before and after the target (A_p_ > P_p_) to isolate top-down auditory attention. This was anticipated to be high in the attentive listening phase (A_p_), when subjects were actively awaiting the target, and lower in the passive listening phase (P_p_) after the target. The auditory input during A_p_ and P_p_ was equivalent. Importantly, activity associated with motor responses did not affect the critical contrast between A_p_ and P_p_, as the response occurred after each trial. Further, the decision about whether a pitch change had occurred, which could evoke implicit or preparatory motor control, occurred during the elongated target period, and so was isolated from the active or passive listening phases.

Extended trial (40 s) and target (10 s) durations were necessary to allow the activity associated with attentional state and target detection to be clearly separated. Longer conditions such as these can result in reduced signal in standard univariate contrast analyses (e.g. Visscher et al., 2003) due to the attenuation of the repeated neural signal and the transient pattern of activation associated with attentional reorienting occurring within each condition block. Multivariate techniques such as Independent Component Analysis (ICA) are able to decompose the BOLD signal into multiple different components, and therefore isolate different sources of variation in the data that may obscure task-evoked signal over extended durations. ICA is therefore more suited to the current elongated design.

We also recreated the experimental conditions and attentional requirements in an analogous visuospatial search task in a different sample of subjects to confirm the prediction that visual top-down attention would evoke the activation of the DAN, including FEF, SPL and MFG. We hypothesized that the auditory task would activate a top-down attention network that was distinct from the DAN, whereas the full DAN would be activated during the visual task.

### Subjects

Forty healthy right-handed volunteers took part in this study: 20 in the auditory task (9 female, mean age 29.7, range 22 to 52) and 20 in the visual task (10 female, mean age 25.7, range 22 to 45). All participants had normal vision or corrected vision (via contact lenses or MRI compatible glasses) and reported no hearing problems. The study was conducted in accordance with the guidelines of Imperial College Research Ethics Committee, and written consent was obtained from all volunteers before their participation. Participants were screened for contraindications to MRI, and were excluded on the basis of color blindness, hearing difficulties or previous psychiatric or neurological disorders.

### Auditory search task

Subjects were played 6 different 40 second (s) stereo naturalistic background sounds (e.g. of a busy high street, or tropical ‘dawn chorus’) obtained from the BBC Sound Effects Library binaurally (Fig. 1). The background sounds were complex, with lots of potential distractors, so that continuous attentive listening was required to identify the task-relevant sounds (Leech et al., 2009). Subjects were trained beforehand to identify the two target sounds and pitch changes while listening to the background sounds. Subjects were instructed to listen out for a 10 s target sound (either a spoken sentence or a sequence of tones) which was presented unpredictably over the background sounds in 80% of the trials. The subjects' task was to report whether or not they heard a pitch change during the target sound. Subjects were trained beforehand to identify the target sounds and pitch changes while listening to the background sounds. Targets were presented within the middle 30 s of each 40 s background sound, jittered around either early (5–7 s) or late (22–24 s) onset. 75% of the target sounds presented had a 1-second long pitch modulation of 13 semitones. This pitch change was jittered around either early (2 s after target onset) or late (8 s after target onset) positions.

There were 40 trials per subject, split into two blocks of 20 (one each for language and non-language targets). A black screen with the words “LISTEN OUT FOR TONES” or “LISTEN OUT FOR SPEECH” written in red font was presented during the whole trial. An identical target sound was presented at both ears simultaneously, meaning there was no spatial cue involved in the target detection task. The tone target consisted of a repeating diatonic melody of major triad notes. The speech target consisted of a woman's voice reading the sentences “It was not in the least like anything he had ever felt before. It grasped him as definitely and instantaneously as a giant hand might have done” obtained from the Open Source Audio Library (from ‘The Buddhic Consciousness’ by Charles W. Leadbeater). The sentence was specifically chosen to have limited emotional or semantic content.

In order to avoid the motor response affecting the BOLD activation, subjects waited till the end of the trial before being cued to press a button indicating their behavioral response (“RESPOND: was there a pitch change?” lasting 3 s). For their response, subjects were instructed to click with their right hand if they heard the target with a pitch change, with their left hand if they heard the target but not a pitch change, and to make no response if they did not hear the target. As such the chance level of the responses was 33%. This was followed by a 5 second rest period (“PLEASE WAIT, Loading”) between each trial.

To ensure that the background sounds during the A_p_ and P_p_ were approximately equivalent, we calculated and compared a range of summary acoustic measures as in a previous work (Leech et al., 2009). The spectral centroid, standard deviation, skewness and kurtosis were calculated, as were the harmonic energy to noise ratio and the average root mean squared intensity. All of these acoustic measures showed that the background sounds presented in A_p_ and P_p_ conditions were equivalent in terms of acoustic complexity.

### Visual search task

The visual search task was designed to replicate the auditory search task in as many dimensions as possible except in the visual rather than auditory domain. The overall timings and number of each type of condition were matched across the two tasks. Instead of naturalistic background sounds, color video footage of naturalistic scenes (e.g. of shoppers on a busy street or a complex underwater scene) obtained from a variety of online sources was used. The task was to detect a 1 s color change of the target stimuli (from red to green). Targets consisted of either a red rectangle or a written sentence (“It was not in the least like anything he had ever felt before. It grasped him as definitely and suddenly as a giant hand might have done”) in red words presented in the same location one at a time for 0.4 s each word. Targets appeared in two possible locations on the screen (top-left or bottom-right) with the same onsets as in the auditory task. Subjects (n = 20, unpaired sample) were instructed to respond at the end of each trial as in the auditory task. Visually complex moving scenes were used so that continuous top-down monitoring was required in order to detect the target.

### MRI data acquisition

MRI data were obtained using a Philips Intera 3.0 T MRI system with an 8-element phased array head coil and sensitivity encoding. High-resolution (1 mm × 1 mm × 1 mm) T1-weighted whole-brain structural images were obtained for each participant to allow accurate spatial registration of the functional images. Functional MRI data were acquired using an echoplanar imaging (EPI) sequence. Continuous data acquisition was used to collect whole-brain images in 44 axial slices with a slice thickness of 3.5 mm, and a repetition time (TR) of 3 s (TE = 45 ms, FOV = 220 × 143 × 190 mm). A total of 670 whole brain functional images were acquired for each subject, split into two runs of 335 images. Paradigms were programmed using Matlab Psychophysics toolbox (Psychtoolbox-3 www.psychtoolbox.org) and stimuli presented through an IFIS-SA system (In Vivo Corporation). Responses were recorded through a fiber optic response box (Nordicneurolab, Norway), interfaced with the stimulus presentation PC running Matlab.

### FMRI image analysis

Standard preprocessing was carried out using FSL (Smith et al., 2004; FMRIB's Software Library, www.fmrib.ox.ac.uk/fsl). Image pre-processing involved realignment of EPI images to remove the effects of motion between scans, spatial smoothing using a 5 mm full-width half-maximum Gaussian kernel, pre-whitening using FILM and temporal high-pass filtering using a cut-off frequency of 1/50 Hz to correct for baseline drifts in the signal. FMRIB's Nonlinear Image Registration Tool (FNIRT) was used to register EPI functional datasets into a standard MNI space using the participant's individual high-resolution anatomical images.

### General linear model

For both the auditory and visual experiments, four variables were entered into a general linear model: attentive listening/viewing phase (A_p_), target, passive listening/viewing phase (P_p_) and response. The model included the full duration of the A_p_, P_p_, target and response conditions. A synthetic hemodynamic response function was convolved with each explanatory variable and its first temporal derivative was included to account for variability in the hemodynamic delay function. This ensured that there was adequate time in even the shortest conditions (5 s) to resolve the hemodynamic response and allowed adequate comparison of the model to the BOLD and ICA timecourses. The 5 s rest period following each trial was the implicit baseline. To investigate activations due to top-down attention, we contrasted BOLD images acquired during A_p_ with those during P_p_ (A_p_ > P_p_). Due to the jittered target onset, in a minority of the trials (10%) the A_p_ and P_p_ durations were 5 s long, though the average durations were 20.7 s and 16.1 s respectively.

### Univariate subtraction analysis

Mixed effects analysis of session and group effects was carried out using FLAME (FMRIB's Local Analysis of Mixed Effects; Beckmann et al., 2003). Final statistical images were thresholded using Gaussian Random Field based cluster inference with a height threshold of Z > 2.3 and a cluster significance threshold of p < 0.05. This resulted in statistical maps of voxels significantly activated by the task and a separate map of voxels showing a relative deactivation on task.

#### Univariate region of interest (ROI) analysis

We created three spheres of 10 mm radius centered at DAN subregions the SPL (23, − 65, 48), the FEF (32, − 10, 48) and the MFG (46, 6, 42) using MNI coordinates obtained from Capotosto et al. (2009) and Dosenbach et al. (2007). We also took the coordinates of the two prefrontal peak voxels from the audio and visual constrained-ICA analyses, the IFG-a (50, 21, 15) and the MFG-v (33, 22, 37) respectively. These spheres were placed over the individual subjects' univariate contrast images of A_p_ > P_p_ and the data from each voxel in the sphere were averaged together.

### Multivariate whole-brain independent components analysis (ICA)

ICA was carried out using Tensorial ICA (Beckmann and Smith, 2005) as implemented in MELODIC (Multivariate Exploratory Linear Decomposition into Independent Components, Version 3.10, part of FSL) which aligns data from each subject in time (not concatenated). Pre-processed data were whitened and projected into a 10-dimensional subspace using Principal Component Analysis. The approach decomposes the whole brain spatio-temporal fMRI data into independent largely non-spatially overlapping components. This is only one possible approach and alternative techniques, such as temporal ICA, make different assumptions about the underlying signal (e.g. that it should be decomposed into largely non-temporally overlapping components), and could, therefore, provide a different perspective (see Calhoun et al., 2012 TICS for a discussion of this). In addition, the results of the ICA are affected by the number of components sought. The choice of 10 dimensions was made based on a previous work (Leech et al., 2012) but additional dimensionalities were also investigated (see Fig. 4). ICA components consist of a spatial map and a single timeseries which describes the change in the activity of this component over time. To assess which components were modulated by task, we entered relevant ICA timeseries as the dependent variable with our general linear model. We corrected for the increase in the family-wise error from making multiple comparisons by using a Bonferroni correction for the 10 components calculated. All p values in the main text are Bonferroni corrected unless stated otherwise. Spatial maps of each independent component were created using a > 0.5 threshold for the Gaussian mixture model.

**Fig. 2:**
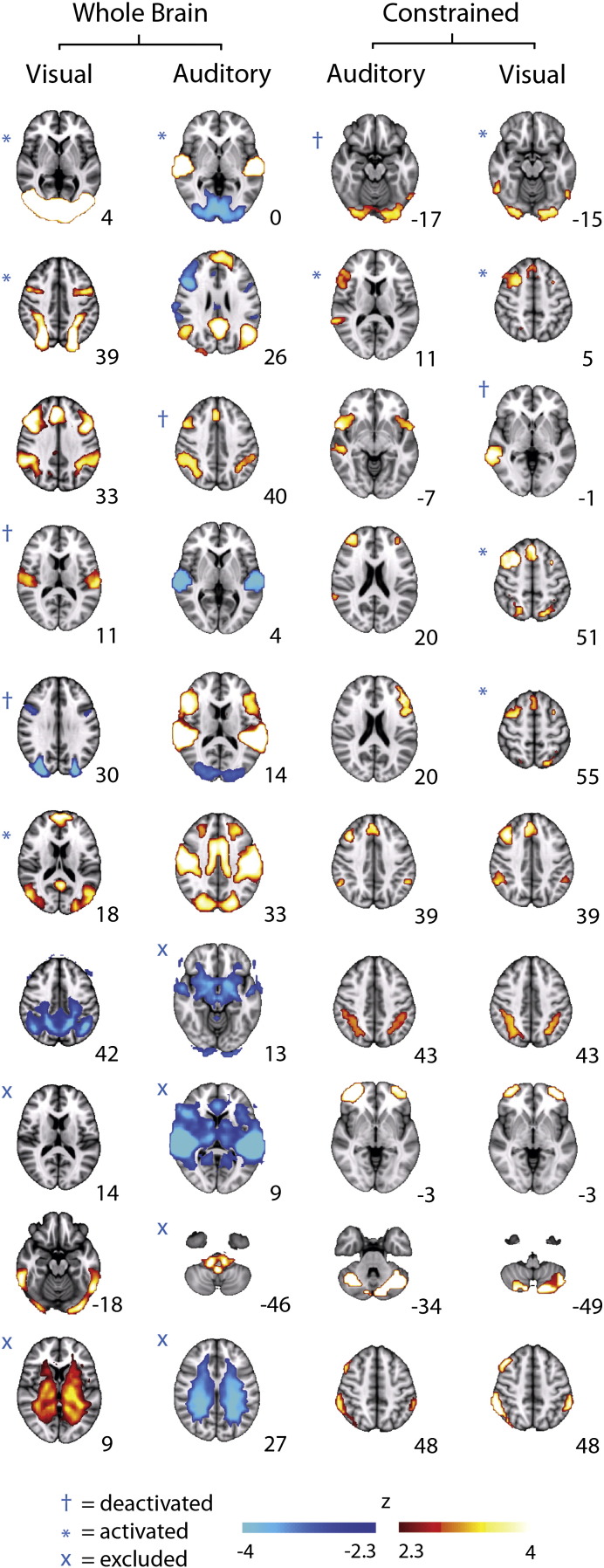
Independent component analysis (ICA) results. (left) Whole-brain ICA derived from both the visual and auditory datasets. For the visual dataset, five components were modulated by an attentional load (attentive phase > passive phase: A_p_ > P_p_, blue symbols, p < 0.01 Bonferroni corrected), including the typical dorsal attentional and visual networks. Two components were deemed to be artifactual and were excluded from subsequent analysis as in Smith et al. (2009). From the ten auditory components, two were modulated by an attentional load. The whole-brain ICA fronto-parietal spatial map from the auditory data (third from top) was then used to spatially-constrain another ICA on auditory and visual datasets (right). This fractionated the fronto-parietal network into ten sub-networks in each data set. Spatially similar sub-networks were produced in both constrained analyses (bottom axial images). However, different sub-networks were modulated by top-down attention (blue symbols), as revealed by our general linear model (A_p_ > P_p_, p < 0.05, corrected). Numbers refer to MNI152 atlas coordinates along the z-axis. Modulations shown refer to A_p_ > P_p_ contrast. All deactivations in this contrast can equally be interpreted as activations in the reverse P_p_ > A_p_ contrast.

**Fig. 3:**
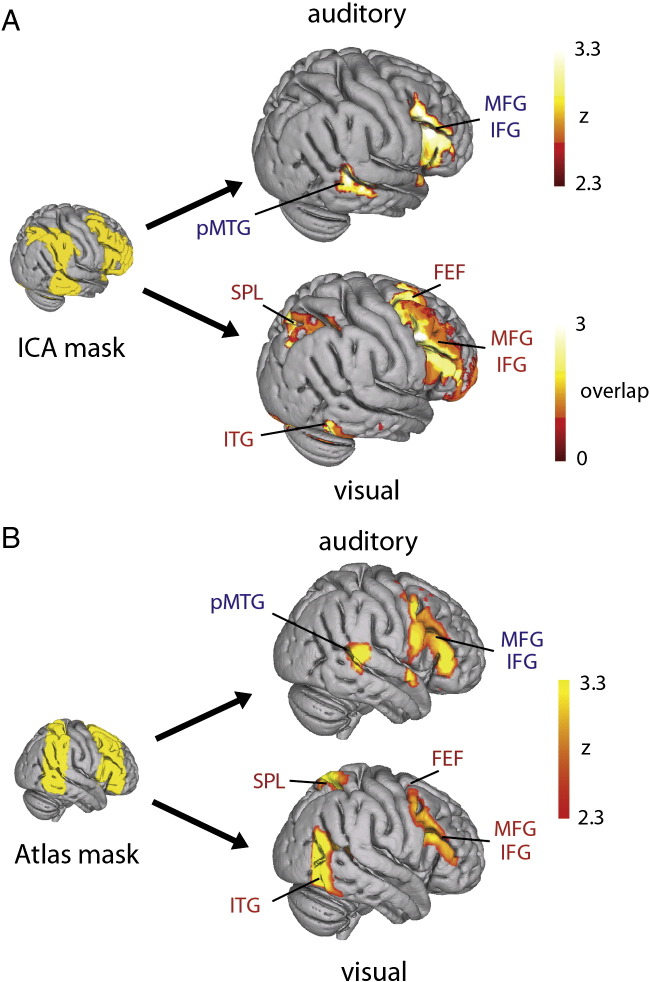
Comparison of auditory and visual top-down attention. Spatially constrained independent component analysis (ICA) of auditory and visual search tasks using functionally (A) and anatomically (B) derived regions of interest (ROI). Both methods revealed similar results. Auditory searching evoked increased activity in middle (MFG) and inferior frontal gyri (IFG) and posterior middle temporal gyrus (pMTG; A_p_ > P_p_, p < 0.05, Bonferroni corrected, single component in both cases). Visual searching (A_p_ > P_p_, p < 0.05, corrected, A: overlap image of 4 components, B: single component) revealed superior parietal lobe (SPL), frontal eye fields (FEF), IFG and MFG activation. Activation of the inferior temporal gyrus (ITG), extending into occipital fusiform gyrus, was observed in both visual analyses. The functional region of interest (ICA mask) was created by binarising the frontoparietal component from a whole-brain ICA of the auditory data. The anatomical region of interest (atlas mask) was created by combining regions of the Harvard–Oxford cortical atlas.

**Fig. 4:**
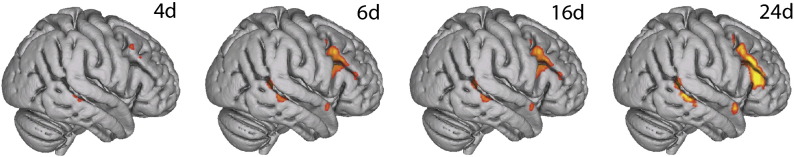
Spatially constrained independent component analysis (ICA) of auditory data at various dimensionalities (4, 6, 16, 24) using functional region of interest: Each analysis revealed similar regions where activity was significantly increased during attentive listening (A_p_ > P_p_, p < 0.05, Bonferroni corrected). The results reported in the text (10 dimensions) were qualitatively robust across all analyses. The increase in activation size with increasing dimensionality (left to right) illustrates how higher-dimensional ICA is able to model noise more accurately by parcellating non-Gaussian variations in the data within additional components. This results in higher confidence in the activation patterns.

The timeseries of these 10 components were assessed for attentional modulation across A_p_ > P_p_. Four components were deemed to be largely artifactual by the standards described by Smith et al. (2009): components were classed as artifacts and excluded from further analysis if the majority of voxels were in white matter, ventricles or outside the brain. Two of the remaining six whole-brain components were modulated by attentional load (i.e. showed significantly increased activation in the contrast of A_p_ > P_p_ or P_p_ > A_p_, Fig. 2). One component showed increased activity in bilateral auditory regions during attentive listening with an associated deactivation in anticorrelated visual areas (p < 0.01, corrected). A right-hemisphere dominant fronto-parietal component was also modulated by attention, but was overall less active during attentive listening (p < 0.01, corrected). This network was large and functionally heterogeneous, including both canonical DAN and VAN regions, which may be anti-correlated during top-down attention (Corbetta et al., 2008), and parts of primary visual regions that may be deactivated during attentive listening (Langner et al., 2011). It is therefore possible that the deactivation observed across this whole-brain network was driven by smaller, localized subregions of the network, which may or may not have overlapped with the DAN. The whole-brain ICA cannot tell us which subregions of this network were driving the deactivation. We therefore performed a spatially restricted ICA to fractionate the whole-brain network and suggest candidate subnetworks that are most important for task modulation. This two-step ICA can be thought of as akin to running statistics such as t-tests subsequent to an ANOVA to determine which factors are driving the result.

### Spatially restricted ICA

In addition to the whole-brain ICA, we used a spatially restricted ICA approach to identify candidate subnetworks that might be modulated during the A_p_ phase. First we defined an ROI and then ran an ICA within this region to decompose it into multiple subcomponents. In principle, similar results to this two-stage approach could be achieved using a single whole-brain ICA at a high dimensionality. However, in practice, gaging the appropriate dimensionality would be difficult given the inherent trade-off between granularity and noise in ICA. In addition, the interpretation of the results would be hampered by multiple comparison problems. The two-stage approach avoids these issues by constraining the ICA to regions that are theoretically interesting. The spatial restriction was done in two ways: (1) using an anatomically defined ROI; and (2) using a functionally defined ROI.

#### Anatomically defined ROI

Based on the previous literature (Corbetta et al., 2008) we generated an anatomically defined mask that covered brain regions thought to comprise both the dorsal and ventral attentional networks. The following regions from the Harvard–Oxford cortical structural atlas were combined: the inferior, middle and superior frontal, temporo-parietal and superior parietal regions: the superior and inferior parietal lobes, angular gyrus and supramarginal gyrus (posterior part), middle temporal gyrus (temporo-occipital part) and inferior, middle and superior frontal gyri. Anatomical probability maps were thresholded at 10% and combined to form a large mask which was resampled into a 4 mm functional space and used to spatially constrain an ICA at 10 dimensions of the auditory and visual task data.

#### Functionally defined ROI

The initial whole-brain ICA of the auditory search task data was used to define a ROI for our dataset in a data-driven way. The whole-brain ICA generated ten components and the right-hemisphere dominant frontoparietal component was selected as a functionally defined ROI. This ROI was then used as a spatial restriction for both auditory and visual datasets.

## Results

### Auditory top-down attention evokes a frontotemporal network

**Fig. 5:**
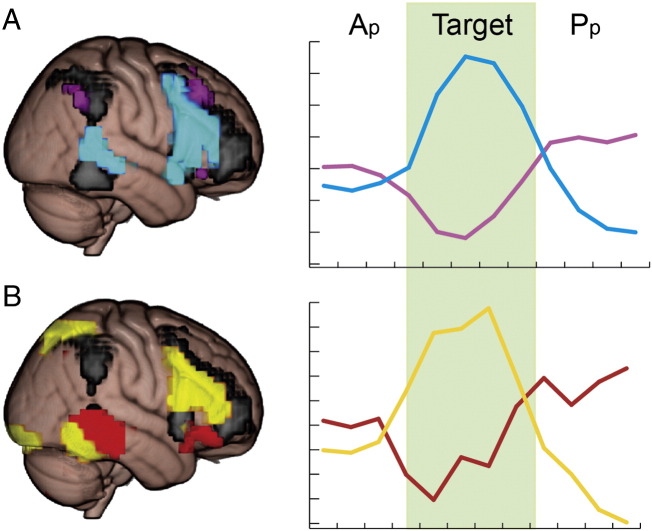
Trial-averaged timecourses taken from the components of spatially restricted independent component analysis (ICA) of auditory (A) and visual (B) tasks. Y-axis displays the relative change in BOLD signal in arbitrary units. X-axis displays the time in TRs (3 s). The signal from each trial was centered around the onset of the target and the BOLD signal from 3 TRs before to 4 TRs after each target was averaged together across trials. A) In the auditory attention task, the component in blue showed a significant activation prior to target presentation (attentive phase; A_p_) compared to the passive phase (Pp). Meanwhile the component in purple, which overlapped with dorsal attention network (DAN) regions, showed no significant difference in the A_p_ > P_p_ contrast (p < 0.05, Bonferroni corrected) and a deactivation during the target phase. B) In the visual attention task, the frontoparietal component in yellow showed a significant activation during A_p_ > P_p_. Another component (in red), which overlapped with our putative auditory top-down attention network (blue), showed a deactivation during A_p_ > P_p_. The spatial mask from the whole brain auditory ICA that was used as a restriction mask for both analyses is shown in black.

The spatially restricted ICAs initially extracted 10 components, although the results were qualitatively similar at multiple dimensionalities (range 4 to 24, Fig. 4). In both functionally and anatomically restricted analyses, only one independent component showed increased activation during attentive listening (Fig. 3, p < 0.05, corrected, and Fig. 5A, blue). This network included right middle and inferior frontal gyri (IFG) and right posterior middle temporal gyrus (pMTG, Table 1). The frontal portion overlapped with the middle frontal regions previously reported to be part of the DAN. The posterior middle temporal region did not overlap with regions of either the DAN or the ventral attention network. Instead, the pMTG region was adjacent to regions reported to be auditory association cortex (Belin et al., 2002). Both restricted ICAs produced multiple fronto-parietal components which overlapped with the DAN (Fig. 2, Fig. 5A, purple), although none of these were significantly modulated by attentive listening (p > 0.34, uncorrected). The functionally restricted ICA also revealed a component with a strong deactivating signal that was largely confined to the visual cortices (Fig. 2). This localized deactivation may have driven the overall deactivating signal found in the whole-brain ICA frontoparietal network. These visual regions were not considered in the anatomically restricted ICA.

**Table 1:** Activation clusters from the spatially constrained independent component analysis of visual and auditory tasks using functional region of interest. MNI atlas coordinates refer to the center of gravity of each cluster. R, Right; L, Left; IFG, inferior frontal gyrus; MTG, middle temporal gyrus; Occ Fusiform, Occipital fusiform gyrus; MFG, middle frontal gyrus; SFG, superior frontal gyrus; FEF, frontal eye fields; and ITG, inferior temporal gyrus.

	MNI			# voxels	Direction
	x	y	z		
Auditory task					
R IFG	50	21	15	346	A_p_ > P_p_
R MTG	61	− 37	2	97	A_p_ > P_p_
L occ fusiform	− 13	− 86	− 20	256	P_p_ > A_p_
Visual task					
R MFG	33	22	37	782	A_p_ > P_p_
R FEF	24	13	52	210	A_p_ > P_p_
L IFG	− 45	32	15	281	A_p_ > P_p_
R IFG	49	20	19	312	A_p_ > P_p_
R SPL	30	− 65	49	170	A_p_ > P_p_
L SPL	− 23	− 67	46	51	A_p_ > P_p_
L occ fusiform	− 10	− 83	− 23	327	A_p_ > P_p_
R occ fusiform	29	− 91	− 17	57	A_p_ > P_p_
L frontal pole	− 41	53	− 1	59	A_p_ > P_p_
R ITG	58	− 52	− 18	55	A_p_ > P_p_
R MTG	62	− 37	− 8	243	P_p_ > A_p_

### Visual top-down attention evokes the DAN

To compare our auditory results with visual attention, which should evoke the DAN, we conducted the same analysis on an analogous visuospatial search task. We used the same spatial masks (both anatomically and functionally derived) to constrain an ICA of the visual task data. Ten subcomponents were extracted, many of which spatially resembled those obtained in the auditory constrained ICA (Fig. 2 — right). In the functional restriction, four components were found to have an increased activation during visual top-down attention (A_p_ > P_p_, p < 0.01, corrected). An overlap image of these components resembled the full DAN, including FEF, SPL and MFG (Fig. 3A). The anatomical restriction (Fig. 3B), revealed only one component that was significantly modulated by attentive viewing (A_p_ > P_p_, p < 0.05, corrected) which resembled the overlap image obtained from the functionally restricted ICA.

The only area of overlap between the visual and auditory networks was within the MFG. The striking differences between the auditory and visual results, obtained from tasks with analogous attentional demands and identical analyses, suggest that separable networks, based around a common MFG core, were mediating top-down attention in each task.

We also repeated the same constrained ICA steps using a functionally derived frontoparietal mask from a whole brain ICA of the visual data which was modulated across A_p_ > P_p_ (Fig. 2 — left). Using this visually-defined network to constrain the auditory data revealed no subnetworks that were significantly active across A_p_ > P_p_. The visually-defined constrained ICA of the visual data revealed two networks that were significantly activated during attentive viewing, with both subnetworks resembling the DAN, as in Fig. 3.

### Deactivation of DAN regions found during attentive listening

**Fig. 6:**
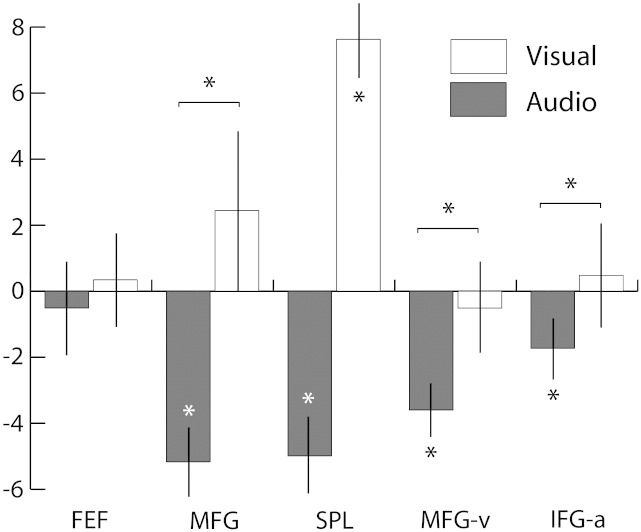
Univariate region-of-interest (ROI) analysis showing changes in activation in dorsal attention network regions during visual and auditory top-down attention. Coordinates for the superior parietal lobe (SPL), middle frontal gyrus (MFG) and frontal eye fields (FEF) were obtained from the literature, while coordinates for MFG-v and IFG-a were the peaks of activation from visual and auditory constrained-ICA results respectively (see Table 1). Y-axis shows normalized regression coefficient values. Error bars represent 95% confidence intervals and asterisks refer to the significance at the 95% level.

The univariate ROI analysis revealed a significant deactivation of two core regions of the DAN, the SPL and the MFG, during attentive listening (A_p_ > P_p_, p < 0.05, d.f. 40, Fig. 6). In contrast, attentive viewing was associated with a significant increase in activation when compared to baseline (SPL) and attentive listening (SPL and MFG, p < 0.05, d.f. 37). This pattern was also observed when the peaks from the visual and audio constrained-ICA analyses were used as ROIs (MFG-v and IFG-a respectively), suggesting that this pattern was not due to a visual attention bias in the coordinates chosen. However, less deactivation was observed in IFG than MFG, which indicates that there may be a superior-inferior gradient (i.e. MFG > MFG-v > IFG-a) for deactivation during auditory attention. No significant modulation across A_p_ and P_p_ was found in the FEF in either modality.

Although the ICA results showed an increased activation in IFG, this was only after parcellating the neural signal into data-driven independent components. The univariate deactivation in the ROI analysis suggests that the dominant neural signal in IFG and MFG is a deactivation during attentive listening. This deactivation may be for several causes, including the inhibition of visual attention during the auditory task. However, the MFG and IFG may be simultaneously involved in suppressing the visual modality and increasing the activation in the auditory modality. Therefore, the signal from the MFG and IFG might be composed of both a dominant deactivating signal for visual attention and another activating signal related to auditory attention. The ICA results suggest that these signals can be split apart and suggest that a portion of the prefrontal signal, namely that which is functionally connected to pMTG, does display an increased activation during A_p_ > P_p_.

A traditional whole-brain univariate contrast image of attentive versus passive listening (A_p_ > P_p_) only revealed a decrease in activation within primary visual cortices during attentive listening (A_p_ > P_p_) after cluster correction (z > 2.3 at p > 0.05) and FDR correction at p < 0.05. A univariate whole-brain analysis of the visual task revealed no regions of significant activation or deactivation during attentive viewing (A_p_ > P_p_, z > 2.3, cluster corrected at p < 0.05). This was despite both analyses being well-powered in terms of subjects (n = 20) and datapoints (mean 256 whole-brain image acquisitions per subject in A_p_ and mean 159 acquisitions in P_p_).

The extended durations of our A_p_ and P_p_ conditions (range 5–25 s) meant that there was an absence of a punctate task, meaning that the multivariate analysis may be better suited than the ROI or whole brain univariate analyses. Transient evoked activity (such as that employed during A_p_) shows a marked attenuation over time (Visscher et al., 2003) and therefore may not be detectable using sustained block designs and univariate statistics. Many overlapping signals might be occurring during each block due to intrinsic coordinated activity and task-irrelevant attentional orienting. The mean signal may therefore be very similar within both conditions, meaning that a univariate comparison A_p_ and P_p_ would yield no, or reduced, activation. Conventional univariate analyses are therefore likely to be suboptimal under circumstances where there are multiple, spatially overlapping neural signals present within a given condition (Beckmann et al., 2005, Leech et al., 2012). In contrast to univariate techniques, ICA is able to produce a better model of the noise in the data and parcellate signals based on their independence from each other. This allows for greater sensitivity to overlapping neural signals (as shown in Fig. 4; Beckmann and Smith, 2004, Leech et al., 2012). We have previously used multivariate ICA to reveal task-modulated neural signals that are not shown by traditional univariate contrasts (Bonnelle et al., 2011, Geranmayeh et al., 2012, Leech et al., 2011, Leech et al., 2012, Sharp et al., 2011).

### Matching for behavior across auditory and visual tasks

**Fig. 7:**
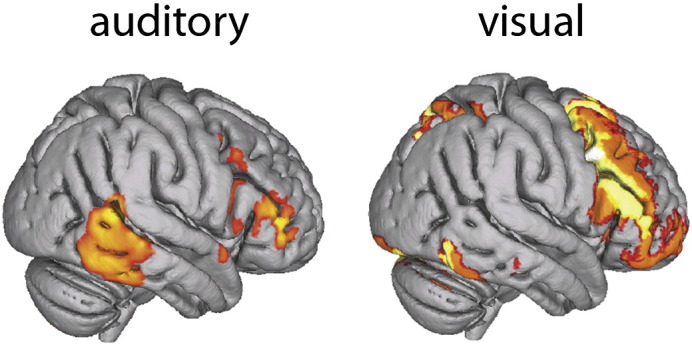
Behaviorally matched independent component analysis (ICA) results: Spatially-constrained ICA using the whole-brain frontoparietal mask was repeated on a behaviorally matched subset of the auditory task group (n = 15, 10 dimensions). One component had a significantly higher activity (p < 0.01, Bonferroni corrected) during attentive listening. This was qualitatively similar to the results using the full group (n = 20) and, was markedly different to the visual task results (right, n = 20, replicated from Fig. 3A).

In the auditory task, subjects were able to detect the targets consistently (average accuracy; 92.3%, n = 17). Behavioral data was not obtained for 3 subjects in the auditory task due to technical problems with the response recording equipment (these subjects were removed from the supplementary behaviorally matched analysis, Fig. 7). In the visual task, subjects were able to detect the presence of the visual targets consistently (average accuracy; 95.2%, n = 20). This is the strongest indicator of attentional engagement during the attentive A_p_ phase, and was matched across the visual and auditory conditions (t(15) < 1.08, p > 0.3).

In the auditory task, subjects were also able to identify the target, i.e. determine if there was a pitch change, significantly above chance (average 74.1%, n = 17). In the visual task, subjects were able to identify the visual targets (i.e. determine if there was a color change) significantly above chance (average 87.5%, n = 20). To confirm that the activation differences we observed were not due to differences in task difficulty, target identification accuracy was matched across the auditory and visual groups by removing the 4 lowest scoring runs from the auditory group (corrected average: auditory 82.1%, n = 15, visual = 87.5%, n = 20, t(33) = 1.31, p > 0.1). A behaviorally matched, spatially constrained ICA of auditory and visual data was then conducted (Fig. 7). This analysis, on a subset of the auditory group, revealed the same results as in the full group, with only a single component (located in MFG and pMTG) significantly activated during attentive listening (p < 0.01, corrected). No activations in SPL and FEF were observed in the performance-matched auditory group.

## Discussion

This study provides evidence that distinct distributed neural networks are activated during auditory and visual top-down attention. The same analyses were conducted on analogous auditory and visuospatial search tasks and implicated separable neural systems. As expected, the activation of the DAN was observed for visuospatial top-down attention (Corbetta et al., 2008). In contrast, we observed significant modulation in a network including MFG and pMTG during non-visual, non-spatial auditory top-down attention. The one area of overlap between the visual and auditory tasks was within the MFG.

One possibility is that the MFG may be a more restricted amodal attentional system that works in concert with modality-specific frontal, parietal and temporal systems during top-down attention. We did not find evidence for DAN activation during attentive listening (observing only deactivation) in either univariate or the more sensitive ICA technique. The contrasting findings in the visual and auditory modalities were statistically robust and were replicated by several analyses (see Fig. 3, Fig. 4, Fig. 6, Fig. 7). Importantly, the pMTG node activated for auditory attention was spatially distinct from regions often implicated in the ventral attention network (VAN), which are usually localized to supramarginal and angular gyri.

Although not previously implicated in auditory top-down attention, the pMTG-MFG network is neurobiologically plausible given that it links ‘executive’ prefrontal regions with temporal regions which are part of the extended higher-level auditory association cortex (Belin et al., 2002, Jamison, 2005). Given the sensorimotor and topographic differences between visual and auditory processing, it is perhaps unsurprising that different functional networks should subserve top-down attention to each modality. It is highly plausible that visual top-down attention should involve regions such as the FEF and SPL with previously reported retinotopic (but not tonotopic) organization.

We observed clear activation of the DAN, including MFG, SPL and FEF, in the visual modality. Recruitment of the SPL and FEF has previously been shown for both spatial and non-spatial visual tasks (Marois et al., 2000). The parietal cortex is heavily involved in spatial awareness, with focal lesions leading to spatial neglect (Behrmann, 2004, Malhotra et al., 2009). As such, it is possible that the activation differences in SPL between the present visual and auditory tasks are due to the non-spatial nature of our auditory task. Previous studies have reported right parietal involvement in auditory spatial localization (At et al., 2011, Shomstein and Yantis, 2006), and our results do not dispute the possibility of a fronto-parieto-temporal network subserving auditory spatial attention. In agreement with Shomstein and Yantis (2006), we observed deactivation of the SPL during attentive listening, further evidence that this region may not be recruited, but rather inhibited, during auditory non-spatial top-down attention. The spatial dimension is intrinsically linked to visual and some forms of non-visual processing, so it is possible that the SPL is active in many forms of top-down attention. However, our isolation of a pMTG-MFG network during non-spatial auditory attention suggests that the DAN does not solely mediate top-down attention as previously suggested (Driver and Spence, 1998, Langner et al., 2011, Macaluso, 2010, Posner and Petersen, 1990). Rather the present results suggest that the networks responsible for top-down attention are flexible to the attended modality.

The FEF and SPL are known to be activated during visual saccades and searching, and in natural visual searching, eye movement control and attention are perhaps inseparable (Nobre et al., 2000). Thus, it is possible that the dorsal DAN activation we observe in our visual task may be due to the increased eye movement required during the attentive phase. Separating visual attention from eye movement was not an aim of the present study. Our visual results are merely confirmatory that natural visual searching elicits the DAN, as has been previously demonstrated (Corbetta et al., 2008, Nobre et al., 2000). In contrast to visual top-down attention, saccades are not necessary for auditory attention, although auditory searching does involve frequent reorienting to the incoming auditory input. It is possible, although impossible to verify, that this reorienting incurs similar cognitive demands in visual and auditory searching. However, the accompanying ocular motor control that is integral to visual orienting is unlikely to be involved in auditory searching. This inherent difference between visual and auditory processing again suggests that the networks required for auditory and visual top-down attention may be separable by necessity.

We also found evidence that these two candidate auditory and visual top-down attention networks are anti-correlated during natural searching to each modality. The ICA timecourses and univariate ROI analyses revealed that during attentive listening we also observe the deactivation of more dorsal frontoparietal regions (Fig. 6). Concomitantly, during attentive viewing, fronto-temporal regions are inhibited while DAN regions are activated (Fig. 5). This suggests that in order to effectively attend to a given modality, networks subserving attention to other modalities may be inhibited.

It is possible that the activation differences found in the A_p_ > P_p_ contrast within each task were driven by the requirements of either the A_p_ or P_p_ conditions. However, the task requirements during P_p_ (i.e. waiting till the end of the trial to respond with a button press) were equivalent in the visual and auditory tasks. As such, the task requirements during P_p_ cannot explain the marked differences between the auditory and visual networks observed. Due to the inherent differences between visual and auditory stimuli, although both tasks required attentional engagement it is not possible to completely ensure that the detection of each target type required equal attentional demand. To control for attentional demand as much as possible we a) used two different targets in each modality, so that differences between any two visual and auditory targets could not determine the result, b) matched target detection accuracy across visual and auditory tasks in all analyses, and c) matched target identification accuracy (i.e. detecting the pitch/color changes) in a behaviorally matched analysis (Fig. 7) which yielded similar results. As such, it is unlikely that differences in target salience were driving the differences between the auditory and visual attention networks identified. Similarly, it is unlikely that behavioral errors account for the difference between visual and auditory results as subjects were able to respond to any contingency, including failure to detect the target or pitch/color changes. This means that the subjects were unlikely to be aware of any errors they committed.

**Fig. 8:**
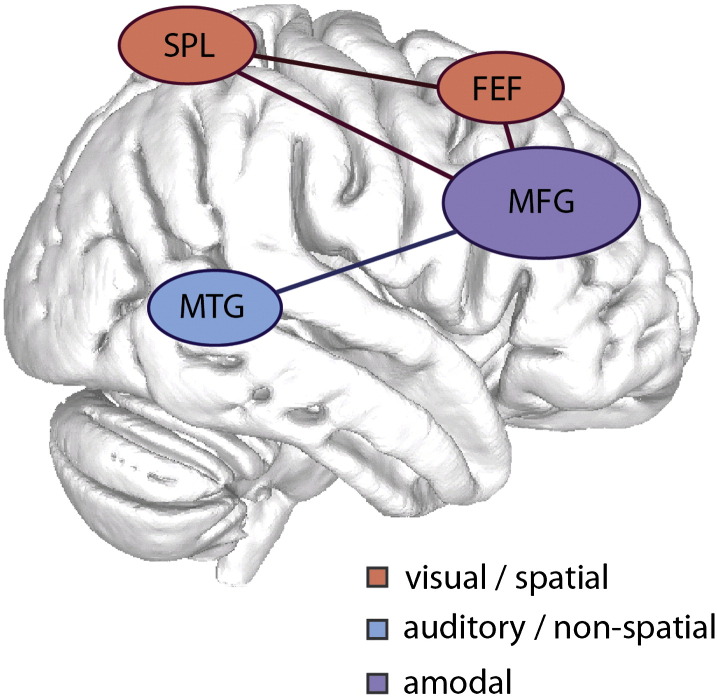
Schematic of our proposed top-down attention system based on our findings from the spatially constrained independent component analyses. An amodal middle frontal gyrus (MFG) is coupled to modality-specific regions, the superior parietal lobe (SPL), the frontal eye fields (FEF) and the middle temporal gyrus (MTG) depending on the attentional demands.

In contrast to the modality specific temporal and parietal regions, we did observe common MFG and IFG increases in activity during top-down attention to both modalities. These regions are involved in a number of higher order cognitive processes, including attention and working memory (Cabeza and Nyberg, 2000). Based on our data and the current literature, we propose that the MFG modulates top-down attention across modalities, but works with separable modal systems depending on the content that top-down attention is oriented towards (Fig. 8). This would explain why SPL stroke lesions cause spatial, but not full auditory neglect (Pavani et al., 2002), why there is an SPL bias in spatial- versus feature-oriented attention (Giesbrecht et al., 2003, Shomstein and Yantis, 2006), and why full DAN activation is not typically reported in speech and music studies (Cabeza and Nyberg, 2000, Hickok et al., 2003, Vigneau et al., 2011, Warren, 2008). Future theoretical neurobiological accounts of cognition should incorporate this more flexible attentional system.
